# The Identification of New Triterpenoids in *Eucalyptus globulus* Wood

**DOI:** 10.3390/molecules26123495

**Published:** 2021-06-08

**Authors:** Ana Lourenço, António Velez Marques, Jorge Gominho

**Affiliations:** 1 Centro de Estudos Florestais, Instituto Superior de Agronomia, Universidade de Lisboa, Tapada da Ajuda, 1349-017 Lisboa, Portugal; analourenco@isa.ulisboa.pt (A.L.); jgominho@isa.ulisboa.pt (J.G.); 2 Instituto Superior de Engenharia de Lisboa, Instituto Politécnico de Lisboa, Rua Conselheiro Emídio Navarro 1, 1959-007 Lisboa, Portugal

**Keywords:** extractives, pentacyclic triterpenoids, oleanane triterpenes, ursane triterpenes, triterpene distribution, GC-MS, ion trap, biorefinery

## Abstract

Eight polyhydroxy triterpenoid acids, hederagenin, (4α)-23-hydroxybetulinic acid, maslinic acid, corosolic acid, arjunolic acid, asiatic acid, caulophyllogenin, and madecassic acid, with 2, 3, and 4 hydroxyl substituents, were identified and quantified in the dichloromethane extract of *Eucalyptus globulus* wood by comparing their GC-retention time and mass spectra with standards. Two other triterpenoid acids were tentatively identified by analyzing their mass spectra, as (2α)-2-hydroxybetulinic acid and (2α,4α)-2,23-dihydroxybetulinic acid, with 2 and 3 hydroxyl substituents. Two MS detectors were used, a quadrupole ion trap (QIT) and a quadrupole mass filter (QMF). The EI fragmentation pattern of the trimethylsilylated polyhydroxy structures of these triterpenoid acids is characterized by the sequential loss of the trimethylsilylated hydroxyl groups, most of them by the retro-Diels-Alder (rDA) opening of the C ring with a π-bond at C12-C13. The rDA C-ring opening produces ions at *m/z* 320 (or 318) and *m/z* 278 (or 277, 276, 366). Sequential losses of the hydroxyl groups produce ions with *m/z* from [M - 90] to [M - 90*y], where y is the number of hydroxyl substituents present (from 2 to 4). Moreover, specific cleavage in ring E was observed, passing from *m/z* 203 to *m/z* 133 and conducting other major fragments such as *m/z* 189.

## 1. Introduction

Terpenoids are secondary metabolites commonly produced by higher plants in different parts of the plant, such as flowers, barks, and roots [1]. These compounds play an essential role in gene expression for plant defense, plant response to stress, and plant interactions: plant–insect, plant–pathogen, and plant–plant interactions [1,2,3]. Terpenoids are also known as isoprenoids, presenting a great variety in number but also in their structure. Their classification can be based on the structural organization of the isoprene units (C5), for example, when constituted by one isoprene unit (hemiterpenoids), two (monoterpenoids), three (sesquiterpenoids), four (diterpenoids), five (sesterpenoids), six (triterpenoids), eight (tetraterpenoids), and others [1]. In particular, triterpenoids are terpenoids composed by a six-isoprene unit skeleton and are derived biosynthetically from the squalene, an acyclic terpenoid with 30 carbons [1]. Over the years, several studies have emphasized the potential of triterpenoids, in particular those with a skeleton of lupane-, oleanane-, and ursane-type that have been strongly associated with a broad spectrum of pharmacological activities—including anti-inflammatory, antiangiogenic, antiviral, antiallergic, antihypertensive, antioxidant, and other activities—and have therefore sparked increasing interest in their potential application in the treatment of cancer [4,5,6,7,8]. More recently, it was found that these compounds, besides their bioactivity, can also be antiviral and thus may have an important role in the treatment of COVID-19 [9]. However, from the commercial point of view, the major challenge of their application is related to a constant and sustainable supply since the identified plants that contain them cannot be seen as feedstock for a large-scale implementation. Thus, it is imperative to continue researching commercial plants that may contain a high content of triterpenoids. This was our motivation driver for studying *Eucalyptus globulus* wood; moreover, it is an important raw material for the pulp and paper industry. With this in mind, eucalypt could be more explored under the biorefinery context by implementing a first stage of extraction (to recover extractives) and a second stage where the extractive-free wood would be used to obtain cellulosic and lignin based-materials. 

*E. globulus* is a valuable pulpwood species in Portugal and Spain due to its fast growth and intrinsic characteristics [10]. *E. globulus* is exploited in short rotation coppices, and it presents a chemical content with low lignin and extractives content comparatively to other *Eucalyptus* species [11,12]. Despite the low content of extractives, they can range from 4% to 6% [10], but in older trees, the values can reach near to 10% [12]. Their composition (in particular the lipophilic extracts) has been extensively studied due to their negative influence in the Pulp and Paper industry. For example, they can accumulate in pulp and equipment [13,14]. In the lipophilic fraction from *E. globulus* mature wood, ten triterpenoid compounds were identified and quantified for the first time, eight were identified by comparison with standards, and two were tentatively identified by analyzing and comparing their spectra with the other identified triterpenic acids. These compounds can be highly valued for pharmaceutical, medical, and other purposes, which may motivate their extraction before the pulping process.

## 2. Results and Discussion

**Figure 1:**
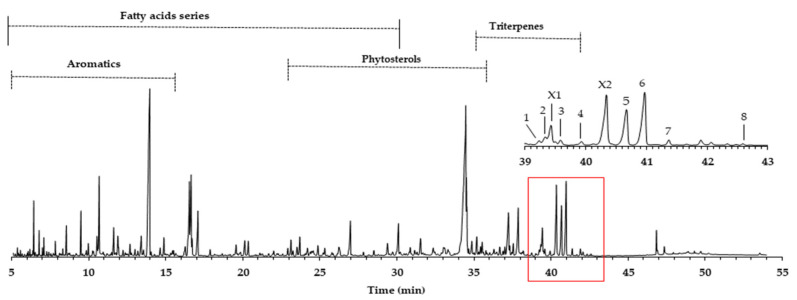
GC-MS chromatogram of *E. globulus* wood dichloromethane extract (attained by QMF). Legend: 1. hederagenin; 2. (4α)-23-hydroxybetulinic acid; 3. maslinic acid; 4. corosolic acid; 5. arjunolic acid; 6. asiatic acid; 7. caulophyllogenin; 8. madecassic acid. X1 and X2 are proposed as (2α)-2-hydroxybetulinic acid and (2α,4α)-2,23-dihydroxybetulinic acid.

The yield of the dichloromethane extracts (DCM), also called lipophilic extracts in eucalypt, is not so high, reaching on average 0.4%, with a minor amount in sapwood (0.2%) and a higher value in heartwood (0.5%). These values are common in eucalypt wood, as reported by other studies: 0.1–0.3% [12] and 0.5% [10]. An example of the GC-MS quadrupole mass filter TIC chromatogram obtained is presented in Figure 1, revealing complexity, either in the number of families or in the number of compounds identified (total 202), as described in Gominho et al. [15]. 

In this work, we focus on the triterpenoids region, where the major number of triterpenes is concentrated in the amplified region of Figure 1, representing 10.4% of the total area and an average of 75.6 mg/g of the DCM extract. Eluting in this retention time zone were common compounds, such as squalene (3.6 mg/g) and betulinic acid (2.9 mg/g). However, other triterpenoids, present in higher amounts and just reported once in *E. globulus* wood by Gominho et al. [15], were identified, such as asiatic acid (peak **6**, 14.5 mg/g) and arjunolic acid (peak **5**, 11.7 mg/g). In minor amounts, (4α)-23-hydroxybetulinic acid (peak **2**, 2.7 mg/g), maslinic acid (peak **3**, 1.6 mg/g), caulophylogenin (peak **7**, 1.6 mg/g), corosolic acid (peak **4**, 1.5 mg/g), hederagenin (peak **1**, 0.8 mg/g), and madecassic acid (peak **8**, 0.1 mg/g) were also identified. Some of these compounds were found in the bark of other eucalypt species in amounts ranging from 130 to 3286 mg/kg of bark [16]. 

**Figure 2:**
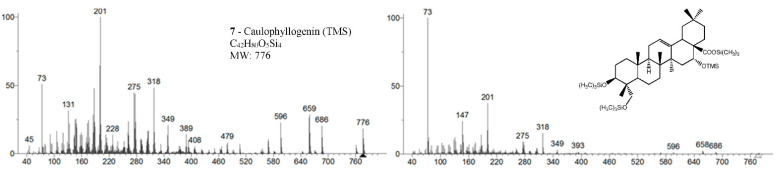
Mass spectra of caulophyllogenin standard (peak **7**, MW TMS 776) from QIT MS (**left**) and QMF MS (**right**). Fragment ions signals (*m/z*): 761 = [M - CH_3_]^+^; 686 = [M - TMSOH]^+.^; 659 = [M - TMSOOC]^+^; 596 = [M - 2 × TMSOH]**^+.^**; 479 = [M – 2 × TMSOH - TMSOOC]^+^; 389 = [M – 3 × TMSOH - TMSOOC]^+^; 147 = [TMSOSi(CH_3_)_2_]^+^; 73 = [TMS]**^+^**; rDA fragments: 318 = [M - DEC*]**^+.^**; 275 = [M - DEC* - TMSOH]**^+^**; 201 = [M - ABC* - TMSOOCH]**^+^**; 131 = [M - ABC* - TMSOOCH - 70 mu]^+^. rDA retro-Diels-Alder fragmentation; C* represents only a portion of ring C.

Samples were analyzed in two GC-MS apparatus with different mass detectors, a 3D quadrupole ion trap (QIT) for mass spectral analysis and identification, and a quadrupole mass filter (QMF) for identification and semi-quantification purposes. The QIT MS detector differs from the fly-through QMF in its capacity to store ions before analysis, providing an increase in sensitivity and signal-to-noise ratio compared to QMF, namely, for fragments of greater mass [17]. For complex and high-mass structures, the detection of fragments closer to the molecular ion is critical to identify and distinguish isomeric compounds, namely stereoisomers. Figure 2 presents the MS spectrum of caulophyllogenin (peak **7**) from the QIT and the QMF. As can be observed, the relative intensities of *m/z* peaks are quite different, with a higher sensitivity for the QIT (left). Although the similarity and the presence of the same mass peaks are evident, important high mass fragments and molecular ion in QIT are more intense, more distinct from the background noise, and base peak mass is 201 rather than the natural and characterless 73. However, QIT is not appropriate for quantification because the detector limits the ion concentration in the detector to avoid ion-molecule reactions. 

The pentacyclic polyhydroxylated triterpenoids, numbered here from 1 to 8 (Figure 1, Table 1), present in the eucalypt samples were identified by comparing their mass spectra and GC retention time (RT) with standards. The retention time was essential for accurate identification because the similarity in skeletons and substituents generate similar mass spectra. Therefore, the GC elution order of these triterpenoids was as follows: hederagenin (**1**), (4α)-23-hydroxybetulinic acid (**2**), maslinic acid (**3**), corosolic acid (**4**), arjunolic acid (**5**), asiatic acid (**6**), caulophyllogenin (**7**), and madecassic acid (**8**). This sequence is related to the following three factors: (i) the molecular weight of the compounds, including the trimethylsilyl groups (688 in compounds **1**, **2**, **3**, and **4**; 776 in compounds **5**, **6,** and **7**; and 864 in compound **8)**; (ii) the number of hydroxyl substituents (OH), excluding the COOH, with the same order, namely two, three, and four; (iii) the E-ring relative position of the methyl groups, that can be both in carbon C20 or apart in anti-configuration at C19 and C20, which has implications in the steric hindrance of equatorial and axial positions and in the planarity of the molecule, thus influences the elution time, i.e., ursane-type skeletons have higher RT than oleanane-type ones [18]. This is the case of corosolic acid (**4**), which has the two methyl groups apart in C19 and C20 and thus has a higher RT, comparatively, to maslinic acid (**3**); the same RT relationship can be observed between arjunolic (**5**) and asiatic acids (**6**).

**Figure 3:**
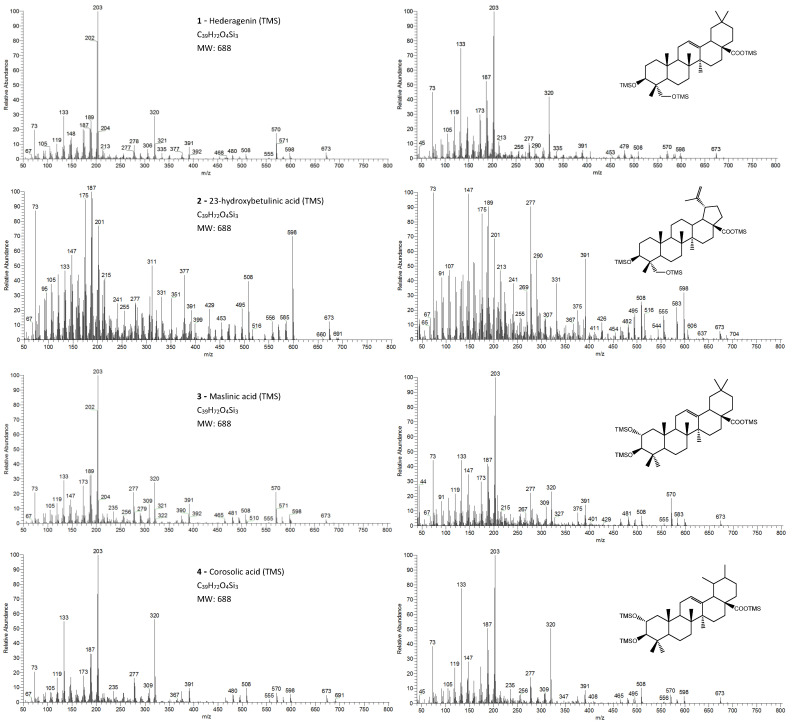
The EI/MS TMS mass spectra of the triterpenoid acid standards (**left**) with 2 hydroxyls (attained by QIT MS): hederagenin, (4α)-23-hydroxybetulinic acid, maslinic acid, corosolic acid ([M]**^+.^** = 688) and the correspondent mass spectra in the *E. globulus* wood sample (**right**). Fragment ions signals (*m/z*): 691 = [M - CH_3_ + H_2_O]^+^; 673 = [M **-** CH_3_]^+^; 598 = [M - TMSOH]**^+.^**; 583 = [M - CH_3_ - TMSOH]^+^; 570 = [M - TMSOOCH]**^+.^**; 508 = [M – 2 × TMSOH]**^+.^**; 495 = [M - TMSOH - TMSOCH_2_]**^+^**; 481 = [M - TMSO - TMSOOCH]+ or [M - TMSOH - TMSOOC]**^+^**; 480 = [M - TMSOH - TMSOOCH]**^+.^**; 391 = [481 - TMSOH]**^+^**; 148 = [TMSOSi(CH_3_)_2_ + H]**^+.^**; 147 = [TMSOSi(CH_3_)_2_]**^+^**; 73 = [TMS]**^+^**; rDA fragments: 320 = [M - ABC*]**^+.^**; 278 = [M - DEC* - TMSOH]**^+.^**; 277 = [M - DEC* - TMSOH - H]**^+^**; 235 = [277 - 42 mu]**^+^**; 203 = [M - ABC* - TMSOOCH]**^+^**; 189 = [M - AB + H]**^+^**; 175 = [278 - 103 mu]**^+^**; 133 = [203 - 70 mu]**^+^**; 119 = [133 - CH_3_ + H]**^+^**. rDA: retro-Diels-Alder fragmentation (Figure 6); C* represents only a portion of ring C; mu mass units.

**Figure 4:**
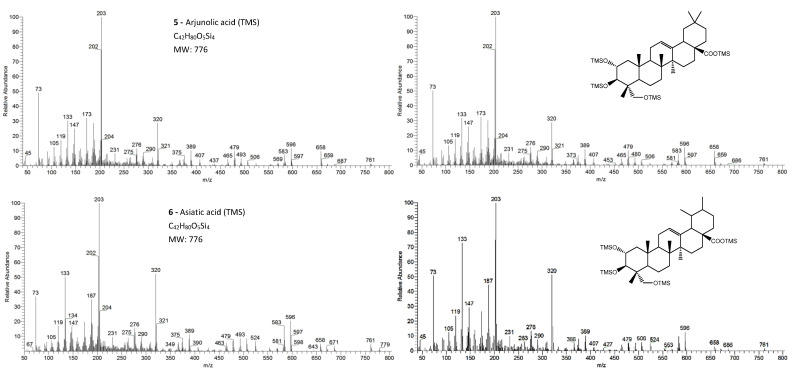
The EI/MS TMS mass spectra of the triterpenoid acid standards (**left**) with 3 hydroxyls (attained by QIT MS): arjunolic acid and asiatic acid (M**^+.^** = 776) and the correspondent mass spectra in the *E. globulus* wood sample (**right**). Fragment ions signals (*m/z*): 779 = [M - CH_3_ + H_2_O]^+^; 761 = [M - CH_3_]^+^; 686 = [M - TMSOH]**^+.^**; 671 = [M - CH_3_ - TMSOH]^+^; 658 = [M - TMSOOCH]**^+.^**; 596 = [M - 2 × TMSOH]**^+.^**; 583 = [M - TMSOH - TMSCOH_2_]**^+^**; 568 = [M - TMSOH - TMSOOCH]**^+.^**; 506 = [M - 3 × TMSOH]**^+.^**; 493 = [M - 2 × TMSOH - TMSCOH_2_]**^+^**; 479 = [M - 2 × TMSOH - TMSOOC]**^+^**; 389 = [M - 3 × TMSOH - TMSOOC]**^+^**; 147 = [TMSOSi(CH_3_)_2_]**^+^**; 73 = [TMS]**^+^**; rDA fragments: 320 = [M - DEC*]**^+.^**; 276 = [M - DEC* - TMSOH]**^+.^**; 203 = [M - ABC* - TMSOOCH]**^+^**; 366 = [M - DEC*]**^+.^**; 187 = [M - AB + H]**^+^**. rDA retro-Diels–Alder fragmentation; C* represents only a portion of ring C.

**Figure 5:**
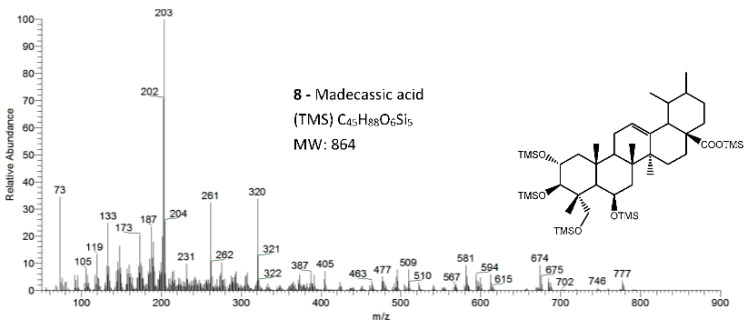
The EI/MS TMS mass spectra of the triterpenoid acid standard with 4 hydroxyls, madecassic acid (Mw = 864, attained by QIT MS). Fragments ions signals (*m/z*): 777 = [M - CH_3_ - TMSOH + H_2_O]^+^; 774 = [M - TMSOH]**^+.^**; 684 = [M - 2 × TMSOH]**^+.^**; 594 = [M - 3 × TMSOH]**^+.^**; 581 = [M - 2 × TMSOH -TMSOCH_2_]**^+^**; 504 = [M - 4 × TMSOH]**^+.^**; 147 = [TMSOSi(CH_3_)_2_]**^+^**; 103 = [TMSOCH_2_]**^+^**; 73 = [TMS]**^+^**; rDA fragments: 320 = [M - ABC*]**^+.^**; 261 = [M - DEC* - TMSOCH_2_ - 2 × TMSOH - H]**^+^**; 231 = [261 - (CH_3_)_2_]**^+^**; 203 = [M - ABC* - TMSOOCH]**^+^**. rDA retro-Diels–Alder fragmentation; C* represents only a portion of ring C.

The eight triterpenoid standards were injected individually in the two GC/MS, and their QIT mass spectra are presented in Figure 2, Figure 3, Figure 4 and Figure 5, with the correspondent mass spectra from the eucalypt sample, except for caulophyllogenin (peak **7**, Figure 2) and madecassic acid (peak **8**, Figure 5) due to sample spectrum quality. The mass spectra resemble each other in most aspects, such as the presence of the peaks attributed to the effect of the trimethylsilylation made on -OH and -COOH groups, which produces the mass fragments at *m/z* 73 [TMS]**^+^** and at *m/z* 147 [TMSiOSi(CH_3_)_2_]**^+^** [19]. In some sample spectra, some inevitable noise derived from poor concentration and GC resolution can be observed.

**Figure 6:**
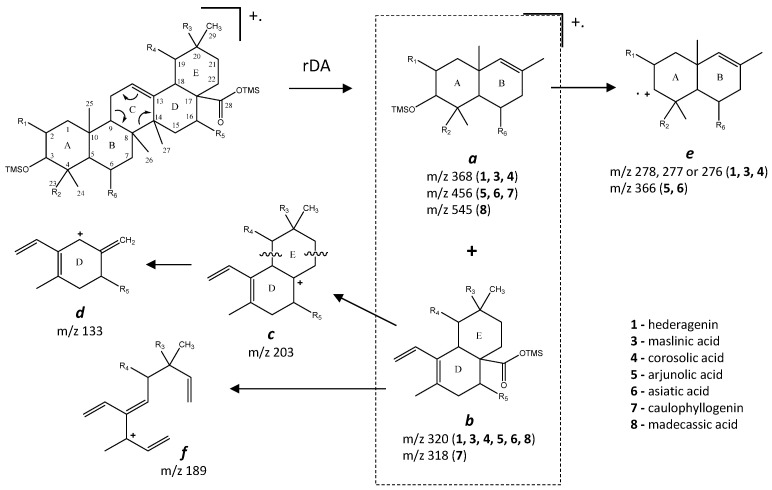
Fragmentation patterns of oleanane and ursane skeleton pentacyclic triterpenoids.

The typical mass ion fragments of oleanane and ursane skeletons include a base peak that, in the QIT, is at *m/z* 203 (73 in QMF) and with the ion at *m/z* 320 from retro-Diels–Alder fragmentation followed by further loss of TMSOOC (mass 117) to *m/z* 203 (discussed further in Figure 6). In the case of the lupane skeleton, such as in (4α)-23-hydroxybetulinic acid (compound **2**, Figure 3), which has no ring π bond, the rDA fragmentation is not favored, and the fragment at *m/z* 320 is weak, thus the base peak is at *m/z* 187, and the loss of trimethylsilanol (TMSOH = 90) groups are more prominent. The molecular ion is very weak or not visible, being the largest mass fragment visible in the spectra, the ion corresponding to the loss of a methyl group [M-CH_3_]**^+^**. The spectra show the characteristic set of ion masses corresponding to the trimethylsilyl derivatives of these polyhydroxyl triterpenic acids, i.e., loss of TMSOOC [M-117] at *m/z* 571, loss of TMSOOCH [M-118] at *m/z* 570, loss of TMSOCH_2_ [M-103] at *m/z* 585, and the serial loss of trimethylsilanol (TMSOH) [M-90] and [M-90-90] with, respectively, *m/z* at 598 and *m/z* 508 [20]. The spectra also show several combination losses of trimethylsilanol (TMSOH) plus a radical: (i) with CH_3_, i.e., [M-90-15] and [M-90-90-15] with fragment ions at *m/z* 583 and 493; (ii) with TMSOCH_2_, such as [M-90-103] with a fragment ion at *m/z* 495; and (iii) with TMSOOC, i.e., [M-90-117] and [M-90-90-117] with fragment ions at *m/z* 481 and 391.

In the QMF analyzer, the spectra of these compounds typically have the same fragments but with a different relative intensity. The base peak is the trimethylsilyl ion at *m/z* 73, the loss of neutral TMSOH fragments is less visible, and the retro-Diels–Alder fragmentation of ring C is more evident with *m/z* 320 more prominent (Figure 2). In the case of caulophyllogenin (peak 7, Figure 2), the characteristic rDA fragment is at 318. According to Isidorov [21], the typical fragments of pentacyclic triterpenoids (such as β-Amyrin and α-Amyrin) are *m/z* 203 and 218, while for the triterpenoids lupeol and betulinic acid, it is *m/z* 189, but for triterpene acids such as ursolic and betulinic acids, it is *m/z* 320. Mathe et al. [19] mention that usually, in a 12-oleanene derivative, the fragment ion *m/z* 203 is more intense than the peak at *m/z* 189, and the opposite occurs for the identical 12-ursane derivative. However, the compounds studied here with a 12-unsaturated oleane skeleton (compounds **1**, Figure 3; and compounds **5**, Figure 4) all present higher *m/z* 203 (at 100%), comparatively, to *m/z* 189 (~30%), with the exception of compound **7** (caulophyllogenin, Figure 2), where the correspondent *m/z* 201 is the base peak. On the other hand, the 12-unsaturated ursane skeleton compounds (**4**, Figure 3; **6**, Figure 4; and **8**, Figure 5) show the same fragmentation profile with minor differences in relative intensity between *m/z* 189/187. 

In the case of asiatic acid (compound **6**), a typical ion-trap mass fragment ion with the strange mass of [M + 3] is visible and presents a mass of *m/z* 779. This mass comes from the ion-molecule reaction between the ion fragment with *m/z* = [M-CH_3_]^+^ and the residual water present in the ion-trap analyzer giving the ion [M-CH_3_ + H_2_O]^+^. This ion mass is typical in TMS derivatives of COOH functional groups [22]. Other fragment masses derived from the same typical ion-trap reactions are visible in the other MS spectra. 

Budzikiewicz et al [23] mentioned that the main MS fragments from pentacyclic triterpenoids with a double bond at C12(13) are derived from retro-Diels-Alder reactions in ring C, resulting in the formation of two fragments: (i) fragment *a* that includes A, B and part of C rings, [M-DEC*]**^+.^**; and another (ii) fragment *b*, involving D, E and part of C rings, [M-ABC*]**^+.^** (Figure 6). Both fragments, can suffer further rearrangements or fragmentation, particularly the fragment *a* that is not noticed in any of the mass spectra attained. This means that it is more likely to be transformed in other ion mass fragments or neutrals: *m/z* 278 [M-DEC*-TMSOH]**^+^**, *m/z* 175 [M-DEC*-TMSOH-103]^+^ and by an abundant ion fragment *c* with *m/z* 203 [M-DEC*-TMSOOC]**^+^**, followed by a mass fragment with *m/z* 133 (*m/z* 203-70 mass units, species *d*). Two Tables are including in Table A1 and Table A2 with the information of these fragments for all the compounds and their mass where slightly different in about 1 or 3 mass units between each compound. The fragment *b*, corresponds to *m/z* 320 for hederagenin (**1**), maslinic acid (**3**), corosolic acid (**4**), arjunolic acid (**5**), asiatic acid (**6**) and madecassic acid (**8**) or *m/z* 318 for caulophyllogenin (**7**). The fragment *b* can also be further splitted into *m/z* 203, 189, 133 as shown in Figure 6.

**Figure 7:**
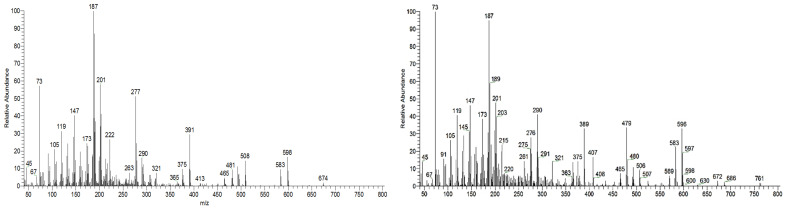
Massa spectra of unknown triterpenic acids X1 (**left**) and X2 (**right**) extracted from a sample run, attained by QIT MS.

The triterpenic acids X1 and X2 accounted for 5.0 mg/g and 13.5 mg/g, respectively, in the samples studied. The triterpenoids X1 and X2 (Figure 1) could not be identified by comparison with standards due to the commercial unavailability of the possible targets. Nevertheless, a tentative identification can be made by analyzing and comparing their spectra (Figure 7) with the other identified triterpenic acids. Both spectra show the characteristic set of ion masses corresponding to the serial loss of trimethylsilanol (TMSOH), [M-90*y], with *m/z* 686 (y = 1), *m/z* 596 (y = 2), and *m/z* 506 (y = 3) for X2, and *m/z* 598 and *m/z* 508 for X1 [20].

X1 (MW TMS 688) shows combination losses of trimethylsilanol plus (i) CH_3_ (15 mu), i.e., [M-15] and [M-15-90*y], with fragment ions at 673, 583 (y = 1) and 493 (y = 2); and (ii) with TMSOOC (117 mu), giving the fragment ions at 481 = [M-117-90*y] with y = 1 and 391 with y = 2. No ions are present for the combination loss of TMSOH with TMSOCH_2_ (103 mu), i.e., no *m/z* ions at 585 = [M-103] and 485 = [M-103-90] are present, in contrast with triterpenes **1**, **2**, **5**, **6,** and **8**, all of them with an OH substituent at C23, which means that in X1, none of the OH substituents are linked to a methylene.

X2 (MW TMS 776) shows combination losses of trimethylsilanol plus (i) CH_3_ (15 mu) thus [M-15] and [M-90*y-15], producing the fragment ions at *m/z* 761, 671 (y = 1), and 581 (y = 2); (ii) TMSOCH_2_ (103 mu), thus [M-90*y-103], generating the fragment ions at *m/z* 583 (y = 2) and 493 (y = 2); and (iii) TMSOOC (117 mu), i.e., [M-90*y-117] with fragment ions at *m/z* 569 (y = 1), 479 (y = 2), and 389 (y = 3). Therefore, the loss of 103 mu means that one of the OH substituents in X2 is linked to a methylene.

None of the triterpenes X1 and X2 show the loss of the neutral carboxy group (TMSCOOH, 118 mu), with no peaks at m/z 570 and 658 for X1 and X2, respectively. Both spectra lack the distinctive and noticeable ion from retro-Diels–Alder fragmentation at m/z 320, presenting instead the mass 321, and both spectra present the general fragmentation features of the trimethylsilylated polyhydroxy triterpenic acids, compounds from 1 to 8, namely the features of (4α)-23-hydroxybetulinic acid, with peak base at m/z 187, i.e., X1 and X2 share the same skeleton with one OH substituent difference, with a MW TMS of 688 for X1 and 776 for X2. Both triterpenes present GC retention times according to the molecular weight and a number of OH substituents of 2 and 3, respectively.

The lack of an rDA characteristic fragment, i.e., the absence of [M-118], peak base at 187, and the characteristic fragment ions present mean that the X1 and X2 triterpene skeleton does not have the characteristic unsaturation, the π bond, at C12-C13. The loss of TMSOOCH can be favored by the presence of the π bond at C12-C13 and by the reduction of energy by the combination of π bonds in the fragment ion. The change of the π bond position and the presence of a more rigid five-carbon E-ring should energetically justify the disadvantage of the loss of TMSOOCH and the very weak presence of *m/z* 320. So, triterpenic acids X1 and X2 probably have a lupene carbon skeleton with unsaturation at C20-C29. Another possible alternative would be an oleanene or an ursene skeleton with the π bond at C20-C21. Verardo et al. [24] studied several TMS derivatized triterpenic acids by GC-MS using the same QIT MS detector from Thermo Finnigan and observed for the MS spectrum of 2α,3β,23-trihydroxy-18α,19α-urs-20-en-28-oic a different relative intensity for the mass ion fragments, despite the fact that they are the same. Beyond that, an unsaturated six-carbon E-ring would not justify the relative difference in retention times. The presence of the unsaturation in any ring other than ring-E would cause a fragmentation specificity that is not apparent in the spectrum. Triterpenes X shows the expected most characteristic ion fragments from a lupene skeleton, *m/z* 187, 189, 201, and 203, with a very weak *m/z* 320 [23].

**Figure 8:**
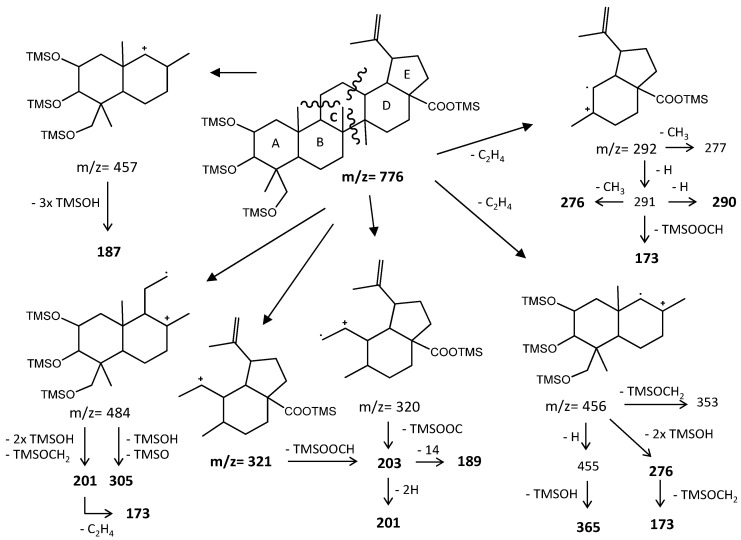
Fragmentation patterns of a lupane skeleton pentacyclic triterpenoid.

**Figure 9:**
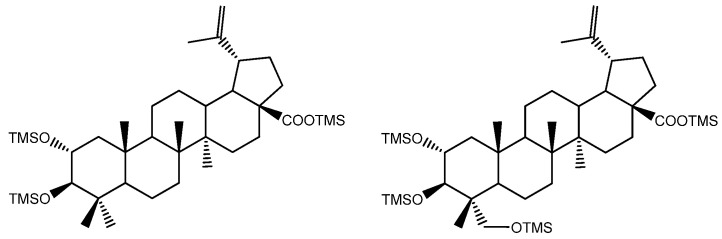
Proposal identification for triterpenic acids X1, (2α)-2-hydroxybetulinic acid and X2, (2α,4α)-2,23-dihydroxybetulinic acid.

Figure 8 represents the fragmentation diagram proposal for triterpenic acid X2. Considering the mass spectra analysis presented, our proposal identification for X1 and X2 is (2α)-2-hydroxybetulinic acid and (2α,4α)-2,23-dihydroxybetulinic acid (hovenic acid) (see Figure 9). 

The importance of these ten pentacyclic triterpenoids is related to their abilities to combat different biological activities. Several researchers have demonstrated the enormous potential of these compounds with pharmacological properties in exhibiting cytotoxicity against a wide range of tumor cells and as anticancer and anti-HIV compounds, as discussed in the following. Hederagenin has anti-inflammatory, antifungal, and anticancer activities [25,26]. Maslinic acid has been found in vegetables and fruits, for example, in leaves and olive fruits. It has been used in traditional Asian medicine, for instance, as an anti-inflammatory against bronchitis and, recently, has gained raised interest since it is a safe molecule for oral consumption, as supported by the in vivo experiments [27]. Its properties are to be seen in a wide range of activities, such as antitumor, antioxidant, cardio, and neuroprotective activities, and it can also help treat cancer, diabetes, and parasitoses [27,28]. Corosolic acid is found in higher amounts in the leaves of Banaba (*Lagerstroemia speciosa*), a native tree of Southeast Asia. The leaves of this plant have been used in traditional medicine to reduce blood sugar levels and help weight loss; more recently, it has been demonstrated to have significant anti-diabetic activity in humans [29,30]. Arjunolic acid started to be isolated from the bark of *Terminalia arjuna* (arjun tree), a famous herbal tree, and later on, from other species such as *Combretum nelsonii* [31]. Arjunolic acid has been used in Ayurvedic medicine as a cardiac tonic, i.e., it has a cardioprotective effect. Studies show that it prevents myocardial necrosis, platelet aggregation, and coagulation, lowering blood pressure, heart rate and decreasing cholesterol levels [31]. Arjunolic acid also plays an essential role in protecting the cells from metal-induced toxicity and is anti-inflammatory, anti-diabetic, antitumor, etc. [31]. Asiatic acid is a natural compound found in fruits and vegetables with many benefits for humans (anti-inflammatory, antioxidant, anti-apoptotic, and anti-carcinogenic) and was reviewed recently by Seo et al. [32]. Asiatic acid was tested in vitro and found to stimulate collagen secretion on human skin, i.e., working as an antioxidant and soothing agent [33,34]. It has been found in extracts from rosemary, lavender, and thyme, but the extracts from *Centella asiatica* have been incorporated into skincare products already on the market [34]. Caulophylogenin was first characterized by Strigina and co-worker [35,36] in *Caulophyllum robustum*, and later on by Becchi et al. [37] in *Chrysanthellum procumbens* with linkages to carbohydrates such as glucose, xylose, and rhamnose, even though it is a less known triterpenoid. Its potential applications were hard to find, but a report mentions an inhibition of inflammatory processes [38]. Madecassic acid has been found in extracts of *Centella asiatica*, and it can stimulate collagen production in human fibroblast, in particular when mixed with asiaticoside, another triterpenic [33]; it belongs to a group of compounds with dermatological and pharmacological activities, such as asiatic acid, asiaticoseide, and madecassoside [34]. Finally, 2,23-dihydroxybetulinic acid was isolated from *Calothamnus quadrifidus* leaves [39] and showed high antibacterial activity.

These pentacyclic triterpenoids exhibit a broad spectrum of therapeutical activities, so it is essential to share the identification and the mass spectra of these compounds and in which plants they are being found.

## 3. Materials and Methods

### 3.1. Sampling

Three *Eucalyptus globulus* trees (40 years) were harvested from a commercial site own by the Portuguese Pulp and Paper industry. The three trees were cut in discs collected at different heights, and each disk was debarked with a chisel. The wood was milled in a Retsch cutting mill (SM 2000) and sieved to attain the 40–60 mesh for analysis. 

### 3.2. Extraction

The milled wood was extracted in a Soxhlet apparatus with dichloromethane (a fairly specific solvent for lipophilic extracts [40] for 16 h. The extracts were recovered after solvent evaporation in a rotary at 40 °C, and then, the residual solvent was evaporated by an N_2_ stream and oven-dried overnight (0.15 bar and 35 °C). The extract was then weighted for yield determination.

### 3.3. Analysis

An aliquot of the DCM extracts (1 mg) and each of the standards (1 mg) were separately dissolved in 120 µL pyridine and then derivatized to trimethylsilyl ethers/esters (TMS) with 80 µL of BSTFA at 60 °C for 30 min. The derivatized samples were injected in two GC-MS apparatus, an Agilent 7890A-5975C MSD and a Thermo Trace Ultra Polaris Ion Trap apparatus from Thermo Finnigan (Austin, TX, USA). Both GC used the same injector type split/splitless, oven temperature program, injector temperature, and column. A splitless injection mode was used in the Agilent, while a split mode was used in the Thermo. The compounds were separated in a high-temperature capillary column Zebron 5HT (30 m × 0.25 mm × 0.1 μm) using helium as carrier gas (1 mL/min flow). The oven program was as follows: 100°C (hold for 1 min), 10 °C/min to 150 °C, 5 °C/min to 200 °C, 4 °C/min to 300 °C, and 10 °C/min to 380 °C (hold for 5 min). Injectors’ temperature was 280 °C, transfer-lines’ temperature was 330 °C (MSD) and 270 °C (QIT), and the temperature was 230 °C for MS sources. The MS electron ionization energy was at 70 eV, and the electronic impact mass spectra (EIMS) were taken over a range of *m/z* 40–950. For the Thermo QIT, a 0.3 mL flow of damping helium was used in the detector and a mean of three mass spectra for each TIC point. The identification of the compounds (as TMS derivatives) was based on comparisons with authentic standards presented here. The compounds were quantified based on their Agilent TIC chromatogram as a percentage of the total chromatogram area. 

### 3.4. Chemicals

The standards: asiatic acid (2α,23-dihydroxyursolic acid, 97% purity), arjunolic acid (2α,3β,4α)-2,3,23-trihydroxyolean-12-en-28-oic acid, ≥95 purity), corosolic acid (2α,3β-dihydroxyurs-12-en-28-oic acid, ≥98% purity), maslinic acid (2α,3β-dihydroxyolean-12-en-28-oic acid, ≥98% purity), and (4α)-23-hydroxybetulinic acid ((3β,4α)-3,23-dihydroxylup-20(29)-en-28-oic acid, ≥98% purity) were purchased from Sigma-Aldrich (Spain, Madrid). The standards: madecassic acid (≥90% purity), caulophyllogenin ((3β, 4α, 16α)-3,16,23-trihydroxyolean-12-en-28-oic acid, ≥98.5% purity), and hederagenin ((3β, 4α)-3,23-dihydroxyolean-12-en-28-oic acid, ≥98.5% purity) were purchased from Extrasynthese (France, Paris). The derivatization reagents (pyridine and N,O-bis-(trimethylsilyl)trifluoroacetamide (BSTFA)) and dichloromethane were acquired from Sigma-Aldrich (Spain).

## 4. Conclusions

The GC/MS analysis of the DCM extract from mature eucalypt wood has led to identifying a group of triterpene compounds representing 10.4% of the total area (75.6 mg/g of the DCM extract). Ten pentacyclic triterpenoids were identified in eucalypt wood extracts and their mass spectra were explained for the first time. Four with an oleanane skeleton (hederagenin, maslinic acid, arjunolic, caulophylogenin), three from ursane-type (corosolic acid, asiatic acid, madecassic acid), and three with a lupane ((4α)-23-hydroxybetulinic acid, (2α)-2-hydroxybetulinic acid and (2α,4α)-2,23-dihydroxybetulinic acid). These compounds are known to have interesting applications, so eucalypt wood extracts are ideal for pharmaceutical development.

## Figures and Tables

**Table 1:**
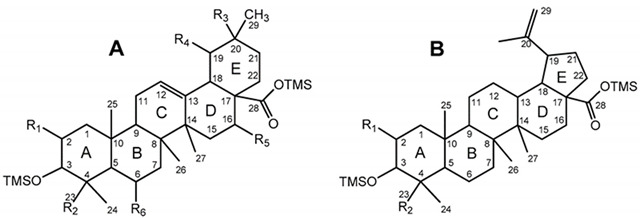
Chemical structure of 1–8 identified trimethylsilylated (TMS) triterpenoids.

Compound	Skeleton	R_1_	R_2_	R_3_	R_4_	R_5_	R_6_	MW	MW TMS
Hederagenin (**1**)	A	H	CH_2_OTMS	CH_3_	H	H	H	472	688
(4α)-23-hydroxybetulinic acid (**2**)	B	H	CH_2_OTMS	-	-	-	-	472	688
Maslinic acid (**3**)	A	OTMS	CH_3_	CH_3_	H	H	H	472	688
Corosolic acid (**4**)	A	OTMS	CH_3_	H	CH_3_	H	H	472	688
Arjunolic acid (**5**)	A	OTMS	CH_2_OTMS	CH_3_	H	H	H	488	776
Asiatic acid (**6**)	A	OTMS	CH_2_OTMS	H	CH_3_	H	H	488	776
Caulophyllogenin (**7**)	A	H	CH_2_OTMS	CH_3_	H	OTMS	H	488	776
Madecassic acid (**8**)	A	OTMS	CH_2_OTMS	H	CH_3_	H	OTMS	504	864

## Data Availability

The data presented in this study are available in the Appendix A section, and any doubt may be referred to the correspondent author.

## Sample Availability

Standards of the compounds and the eucalypt samples mentioned in the manuscript were acquired and collected as described in the material and methods section.

## Appendix A

**Table A1:** Typical fragments formed during mass spectrometry of the TMS spectra of the different standards.

		MW		[M-CH3]^+^	[M-TMSOOCH]^+^	[M-TMSO]^+^	[M-TMSOH]^+^	[M-TMSOH-TMSOOCH]^+^	[TMSOCH_2_]^+^
	Skeleton	TMS	[TMS]^+^	[M-15]^+^	[M-118]^+^	[M-89]^+^	[M-90]^+^	[M-118-90]^+^	
Hederagenin (**1**)	Oleane	688	73	673	570	599	598	480	103
23-hydroxybetulinic acid (**2**)	Lupane	688	73	673	570	599	598	480	103
Maslinic acid (**3**)	Oleane	688	73	673	570	599	598	480	103
Corosolic acid (**4**)	Ursane	688	73	673	570	599	598	480	103
Arjunolic acid (**5**)	Oleane	776	73	761	658	687	686	568	103
Asiatic acid (**6**)	Ursane	776	73	761	658	687	686	568	103
Caulophyllogenin (**7**)	Oleane	776	73	761	658	687	686	568	103
Madecassic acid (**8**)	Ursane	864	73	849	746	775	774	656	103

MW TMS—molecular weight of the trimethylsilylated compounds.

**Table A2:** Specific fragmentation ions derived from the retro-Diels–Alder reaction.

	Skeleton	[M-ABC*]^+^	[M-DEC*]^+^ Do not appear	[M-DEC*-TMSOH*y]^+.^	[M-ABC* -TMSOOCH]^+^	[M-TMSOH-TMSOOCH]^+^	[M-ABC*-70 mu]^+^	[CH_2_OTMS]^+^
Hederagenin (**1**)	Oleane	320^+.^	368^+.^	278^+.^ (when y = 1)	203^+^	480^+.^	133^+^	103^+^
Maslinic acid (**3**)	Oleane	320^+.^	368^+.^	277^+^ (when + H)	203^+^	481^+^	133^+^	103^+^
Corosolic acid (**4**)	Ursane	320^+.^	368^+.^	277^+^ (when + H)	203^+^	480^+.^	133^+^	103^+^
Arjunolic acid (**5**)	Oleane	320^+.^	456^+.^	276^+.^ (when y = 2)	203^+^	568^+.^	-	103^+^
Asiatic acid (**6**)	Ursane	320^+.^	456^+.^	276^+.^ (when y = 2)	203^+^	-	-	103^+^
Caulophyllogenin (**7**)	Oleane	318^+.^	456^+.^	275^+^ (when y = 2)	201^+^	568^+.^	131^+^	103^+^
Madecassic acid (**8**)	Ursane	320^+.^	545^+^	-	203^+^	567^+^	133^+^	103^+^

mu—mass units; y = 1 to 2.
